# Dengue Epidemic in Texas

**Published:** 1885-10

**Authors:** 


DANIEL'S

### Medical ★ Journal,

PUBLISHED MONTHLY AT

AUSTIN, TEXAS.

F. E. DANIEL, M. D., EDITOR AND PUBLISHER

Subscription Price: Two Dollars per Annum in Advance.

Papers for publication in this Journal should be in the hands of the Editor by the 10th of the month preceding the month in which it is wished to appear; they must be original,—never having appeared in print elsewhere, and must be contributed exclusively to this Journal.

Contributions to the department of Original Articles are cordially invited from the professiom of Texas, expecially. What is most desired is Clinical reports of cases, essays and short practical articles on any medical topic; also solicited reports of proceedings of societies, clubs, etc., and items of medical news of general interest to the profession, such as notices of appointments of physicians, removals, changes, marriages, deaths, etc.

### EDITORIAL.

### THE EPIDEMIC OF DENGUE IN TEXAS.

For the third or fourth time within the last few years Austin has been scourged by this distressing, but fortunately, not fatal disease, in epidemic form.

The fact that it is never fatal unless complicated with some other disease, notwithstanding it causes much and intense suffering, loss of time and interruption of business, has perhaps caused both profession and laity to be careless about it, no means whatever being taken to arrest its progress, nor to prevent its introduction. Last year the first case observed in Austin was a lady who had contracted the disease in Galveston. If that case had been isolated, and ordinary precaution taken, no doubt it would have prevented the spread; but her friends visited her, contracted the disease, and thus was it spread over the whole city, The same thing occurred this year. It is believed the first case was in the person of a lady who had been exposed to the infection in Galveston (where it seems to be endemic). As before, no precautions were taken—the disease being generally regarded as infectious, but not contagious. We believe, ourselves, that, though like yellow fever, eminently infectious, and portable, it is not acutely contagious, but contagious only under certain conditions.

This year the city authorities have been engaged extensively in street grading. Though the soil is, for the most part, dry, and containing an abundance of lime, yet in certain locations, and especially in the top earth, there is an abundance of organic matter. Now, just how much this had to do with the development of the epidemic, from the one case brought here, and to contribute towards the "certain conditions," is a matter of speculation. One thing seems to be pretty well agreed upon by the physicians, viz: that the disease is not of local origin, but is always imported. Some of our Austin physicians claim to see more than a mere coincidence between the prevalence of the disease and the street grading, asserting that the disease broke out first, and was most violent in the vicinities where the first and most grading was done. At any rate it seems to us that in view of the fact that to turn up the earth in the hot season is always regarded as detrimental to health, and as there is abundance of room for the suspicion that in the present case it had much to do with the spread of the disease, *if not with its origin*; and remembering it was a factor in the cholera epidemic of Niagara city in 1873, as claimed by Prof. Hamilton and others, it certainly would be the part of wisdom and prudence in our sanitary officers to recommend to the city authorities to prohibit any and all earth excavations during the hot months.

This season the disease has been of a very severe type. There have been no deaths from it, we believe, except in the case of an old man, in whom pneumonia supervened. He died of pneumonia. In some instances the attack is ushered in with quite a severe chill; in others it is preceded a few days by a feeling of *malaise* and loss of appetite. In some—young children, principally—the temperature ran as high as 105–106, and convulsions ensued. Most cases are attended with an erythematous rash; in some, papulæ, in others, resembling urticaria, but mostly a rash resembling measles. This rash appears, for the most part, on the chest, neck and forearms, and on the face. In some the rash appeared before the fever; in others, after, and still on others, there was no rash. Later in the season the disease assumed a hemorrhagic tendency, and epistaxis and bleeding from the gums were frequent. The suffering is said to be dreadful, and persists a long time after the fever declines; and convalescence is exceedingly slow. Nausea is common, but not a constant symptom. We heard of no case of black vomit. Nearly all the physicians had the fever, and at this writing our excellent health officer, Dr. Swearingen, is convalescing from a severe sttack. The disease is no respecter of persons; and this year it has made nearly a clean sweep of the city, sparing neither age, sex nor condition. By-the-by, we have heard very little of it among the negroes. We will look into that. It may be, like its prototype, yellow fever, was once thought to be, of too aristocratic a nature to attack any but whites. While it is generally thought that one attack of dengue affords no protection or immunity, it does seem to us that there is some protection in having had it; for it has been observed in the present epidemic that the few who have not been attalked this season, had the disease last year, or very recently. Some few—our friend Dr. Bennett, the worthy young ex-president of the Travis County Medical Society, among others—have had two attacks this season. When the Doctor came out, his sweatheart didn't know him. Such cases are very rare, however.

It was the intention of some of our Austin physicians to furnish a paper on the subject for this issue of the JOURNAL, but all having been recently down with the disease, are too feeble to make the attempt. We will be able, however, to give our readers one or more scientific papers on the subject in our next number. Some of them are engaged in making examinations and taking notes for that purpose now. It is agreed by all our local physicians that the literature on the subject is very scant and unsatisfactory, and that the disease as described in the books does not at all correspond to the disease as it appears in Texas.

We understand that the epidemic has not been confined to Austin, but is very general, prevailing at nearly all the railroad towns in southern and middle Texas,and as high as Fort Worth. Our esteemed friend, Dr. E. J. Beall, writes us that it is now rife in that city, (Sept. 25.) At present, the disease is on a rapid decline in Austin, simply, we believe, for want of material—all, or nearly all, big and little, old and young, male and female, rich and poor, having had it.

Surely experience, that teacher which alone can teach fools anything, will cause our people to make *some effort* to prevent the yearly occurrence of this dreaded scourge — break-bone fever.

In closing this rambling article, we cannot do better than give our readers the following very original essay on dengue, which was written by that good-natured, cranky local of the defunct *Minute*, M. B. Davis,while the fever was raging in his blood and he was "to busy" to go to bed. We commend it to our readers, who have never felt the delights he there describes, as worthy of a perusal :

THE DENGUE.

"It is the progency of morbid parents. Its dam is a night mare, and its father the blistering witch that strides the blasts, driven from the fens. When the dengue gets hold of a man, he sees his best friends about him, but each one appearr to be an enemy armed to slay him. There is in the prepailing type, the hide of the jim-jams and the hoofs of despair. If one feels inclined to taste the sweets previously—those that Mr. Talmage declares are in store for everybody except himself, let him try a week of the Colorado river dengue and he will be willing to exchange. It appears to the sufferer as if the buzzards fly lower, pausing over him, and gazing down wistfully, hoping to witness his immediate calamity. But in such a case death is not a calamity. It is a bottle of ready relief, from which the feverish hands of the stricken man are eager to tear the wrapper. It is said that the insidious creeping of the virus imparted by the rattle snake, causes much distress. When the dengue gets ready to creep all the creeping things that ever crept, run off into their holes and hide.

Dengue beats the chill of the Brazos bottom because it takes away the power to shake, leaving the tendency; and it beats yellow fever, because the latter kills and dengue never does.

